# Incense Burning is Associated with Human Oral Microbiota Composition

**DOI:** 10.1038/s41598-019-46353-y

**Published:** 2019-07-11

**Authors:** Yvonne Vallès, Claire K. Inman, Brandilyn A. Peters, Laila Abdel Wareth, Abdishakur Abdulle, Habiba Alsafar, Fatme Al Anouti, Ayesha Al Dhaheri, Divya Galani, Muna Haji, Aisha Al Hamiz, Ayesha Al Hosani, Mohammed Al Houqani, Abdulla Aljunaibi, Marina Kazim, Tomas Kirchhoff, Wael Al Mahmeed, Fatma Al Maskari, Abdullah Alnaeemi, Naima Oumeziane, Ravichandran Ramasamy, Ann Marie Schmidt, Henri Vallès, Eiman Al Zaabi, Scott Sherman, Raghib Ali, Jiyoung Ahn, Richard B. Hayes

**Affiliations:** 1 Public Health Research Center, New York University Abu Dhabi, Abu Dhabi, UAE; 2 Department of Biological and Chemical Sciences, The University of the West Indies Cave Hill campus, Cave Hill, Barbados; 3 Department of Population Health, New York University School of Medicine, New York, USA; 4 Pathology and Laboratory Medicine Institute, Cleveland Clinic, Abu Dhabi, UAE; 5 Center for Biotechnology, Khalifa University of Science and Technology, Abu Dhabi, UAE; 6 Biomedical Engineering Department, Khalifa University of Science and Technology, Abu Dhabi, UAE; 7 College of Natural and Health Sciences, Zayed University, Abu Dhabi, UAE; 8 Department of Nutrition, College of Food and Agriculture; UAE University, Al-Ain, UAE; 9 Department of Medicine, College of Medicine and Health Sciences, UAE University, Al-Ain, UAE; 10 Department of Pediatrics, Zayed Military Hospital, Abu Dhabi, UAE; 11 Department of Pathology, Sheikh Khalifa Medical Center, Abu Dhabi, UAE; 12 Heart and Vascular Institute, Cleveland Clinic, Abu Dhabi, UAE; 13 Institute of Public Health, College of Medicine and Health Sciences, UAE University, Al-Ain, UAE; 14 Department of Cardiology, Zayed Military Hospital, Abu Dhabi, UAE; 15 Abu Dhabi Blood Bank, SEHA, Abu Dhabi, UAE; 16 Diabetes Research Program, Division of Endocrinology, Diabetes and Metabolism, Department of Medicine, New York University School of Medicine, New York, USA; 17 NYU Perlmutter Cancer Center, New York, USA

**Keywords:** Metagenomics, Metagenomics

## Abstract

Incense burning is common worldwide and produces environmental toxicants that may influence health; however, biologic effects have been little studied. In 303 Emirati adults, we tested the hypothesis that incense use is linked to compositional changes in the oral microbiota that can be potentially significant for health. The oral microbiota was assessed by amplification of the bacterial 16S rRNA gene from mouthwash samples. Frequency of incense use was ascertained through a questionnaire and examined in relation to overall oral microbiota composition (PERMANOVA analysis), and to specific taxon abundances, by negative binomial generalized linear models. We found that exposure to incense burning was associated with higher microbial diversity (p < 0.013) and overall microbial compositional changes (PERMANOVA, p = 0.003). Our study also revealed that incense use was associated with significant changes in bacterial abundances (i.e. depletion of the dominant taxon *Streptococcus*), even in occasional users (once/week or less) implying that incense use impacts the oral microbiota even at low exposure levels. In summary, this first study suggests that incense burning alters the oral microbiota, potentially serving as an early biomarker of incense-related toxicities and related health consequences. Although a common indoor air pollutant, guidelines for control of incense use have yet to be developed.

## Introduction

Incense burning as part of religious ceremonies, and aromatizing homes and public places is an ancient practice common to Asian and Arabian Gulf countries^1–3^. In the United Arab Emirates (UAE), burning incense is used traditionally to perfume both houses and clothing and is reported to be used at least weekly in >90% of households^4^. Research has shown that the practice of burning incense is a source of ambient air pollution^1^ and that this practice may be related to increased risk of cardiovascular and lung cancer mortality^2,5–7^. Incense smoke contains high concentrations of fine and ultrafine airborne particulates and gaseous pollutants such as carbon monoxide (CO), nitric oxide (NO) and volatile organic compounds (VOCs)^1,8,9^, all of which have also been detected in tobacco smoke^10,11^.

The oral cavity is inhabited by a highly diverse microbial community that plays essential roles in maintaining homeostasis^12,13^. Although known to be one of the most resilient microbiotas of the human body^14^, the diversity, composition and functional potential of the oral microbiota has been shown to be affected by dental health, alcohol intake and tobacco smoke^15–19^. Since both tobacco and incense smoke are composed of similar hazardous agents^9,10^, it could be expected that exposure to incense use would have an impact on the oral microbiota as well.

In this study, we investigated whether exposure to incense burning is associated with changes in diversity, richness and taxa abundances of the oral microbiota. To address this, we have related incense use in the home to the oral microbiota of 303 participants from the UAE Healthy Future Study-Pilot (UAEHFS-pilot). The oral microbiome was measured from mouthwash samples by *16S rRNA* gene sequencing and incense use was assessed by a structured questionnaire, in which frequency of exposure was ascertained.

## Results

### Demographics relative to incense use

Our analysis included 303 subjects who provided a valid informed consent, a completed questionnaire on incense use, and a mouthwash sample from the United Arab Emirates Healthy Future Study-Pilot (UAEHFS-pilot) (Supplementary Fig. S1). The study participants included 6.6% never, 24.1% occasional (one time or less/week), 33.7% frequent (2 to 5 times/week) and 35.6% daily (5–7 times/week) incense users (Table 1). Incense use was reported significantly more often by females then males (p = 0.006). No significant differences in incense use were found with respect to age, education or tobacco smoking habits (p > 0.05).

**Table 1:** Demographic characteristics of the participants according to their incense burning habits.

	Never 20 (6.6)	Occasional^1^ 73 (24.1)	Frequent^2^ 102 (33.7)	Daily^3^ 108 (35.6)	P value
**Incense Use [n (%)]**					
Age, mean (SD)	30.0 (8.6)	33.7 (9.3)	33.9 (10.6)	32.8 (10.9)	0.393^a^
Gender [n (%)]					0.006^b^
Female	5 (25.0)	14 (19.2)	36 (35.3)	48(44.4)	
Male	15 (75.0)	59 (80.1)	66 (64.7)	60 (55.6)	
Smoking habits [n (%)]					0.146^b^
Smoker	2 (10.0)	23 (31.5)	22 (21.6)	36 (33.3)	
Nonsmoker	16 (80.0)	43 (59.0)	72 (70.6)	59 (54.6)	
Unknown	2 (10.0)	7 (9.6)	8 (7.8)	13 (12.0)	
Education [n (%)]					0.251^b^
Secondary school or less	10 (50.0)	35 (48.0)	51 (50.0)	69 (63.9)	
University	8 (40.0)	35 (48.0)	44 (43.1)	32 (29.6)	
Postgraduate	0 (0.0)	2 (2.7)	2 (2.0)	2 (1.9)	
Missing	2 (10.0)	1 (1.4)	5 (4.9)	5 (4.6)	

^1^Occasional users report burning incense in the household one time or less per week.

^2^Frequent users report burning incense in the household 2–5 times per week.

^3^Daily users report burning incense in the household 5–7 times per week.

^a^p values based on Kruskal-Wallis test.

^b^p values based on chi-square test.

### Richness, diversity and overall oral microbial community structure

**Figure 1:**
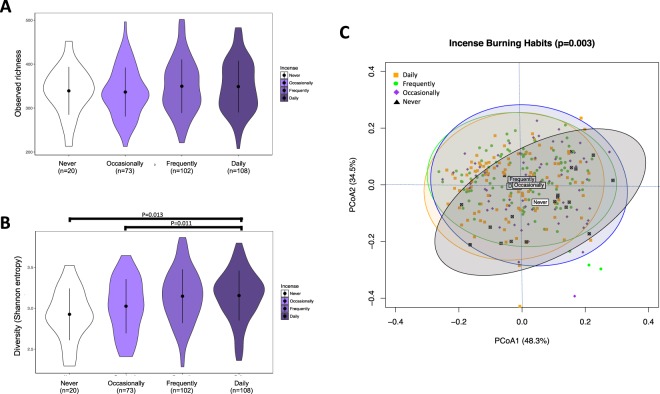
α-diversity and bacterial community structure (β-diversity) of the oral microbiota according to incense burning habits. (**A**) Observed richness and (**B**) diversity (determined by Shannon entropy) of the oral microbiota according to incense burning habits. Indexes were calculated for 200 iterations of rarefied OTU datasets (16738 sequences per sample), followed by calculating the average for each sample. p-values were obtained through multiple linear regression. (**C**) β-diversity was evaluated at the OTU level by implementing PERMANOVA and visualization using plots of Principal Coordinate Analysis (PCoA) of the weighted UniFrac distance matrix. Occasional users reported burning incense in the household one time or less per week, frequent users 2–5 times per week and daily users 5–7 times per week.

After filtering of poor quality sequences and exclusion of low count OTUs, we obtained a dataset of 13,470,071 high quality *16S rRNA* sequences representing 13 phyla, 20 classes, 26 orders, 41 families, 57 genera and 1102 OTUs. Measures of richness (observed) and diversity (Shannon entropy) revealed that diversity of the oral microbiota was significantly increased in daily incense users, compared to never (p = 0.013) and occasional (p = 0.011) users (Fig. 1). Overall oral microbiota structure and composition was also significantly different among the incense frequency use groups, largely driven by differentials between never and daily users of incense (p = 0.003, Fig. 1C), as revealed by Permutational multivariate analysis of variance (PERMANOVA) of UniFrac weighted distances adjusting for age, gender, smoking habits and assay batch. Interestingly, gender (whether modeled as a single factor or via an interaction with incense use) was not significantly associated with microbiota composition (Supplementary Table S1).

### Differences of taxa relative abundances as exposure to incense increases

**Figure 2:**
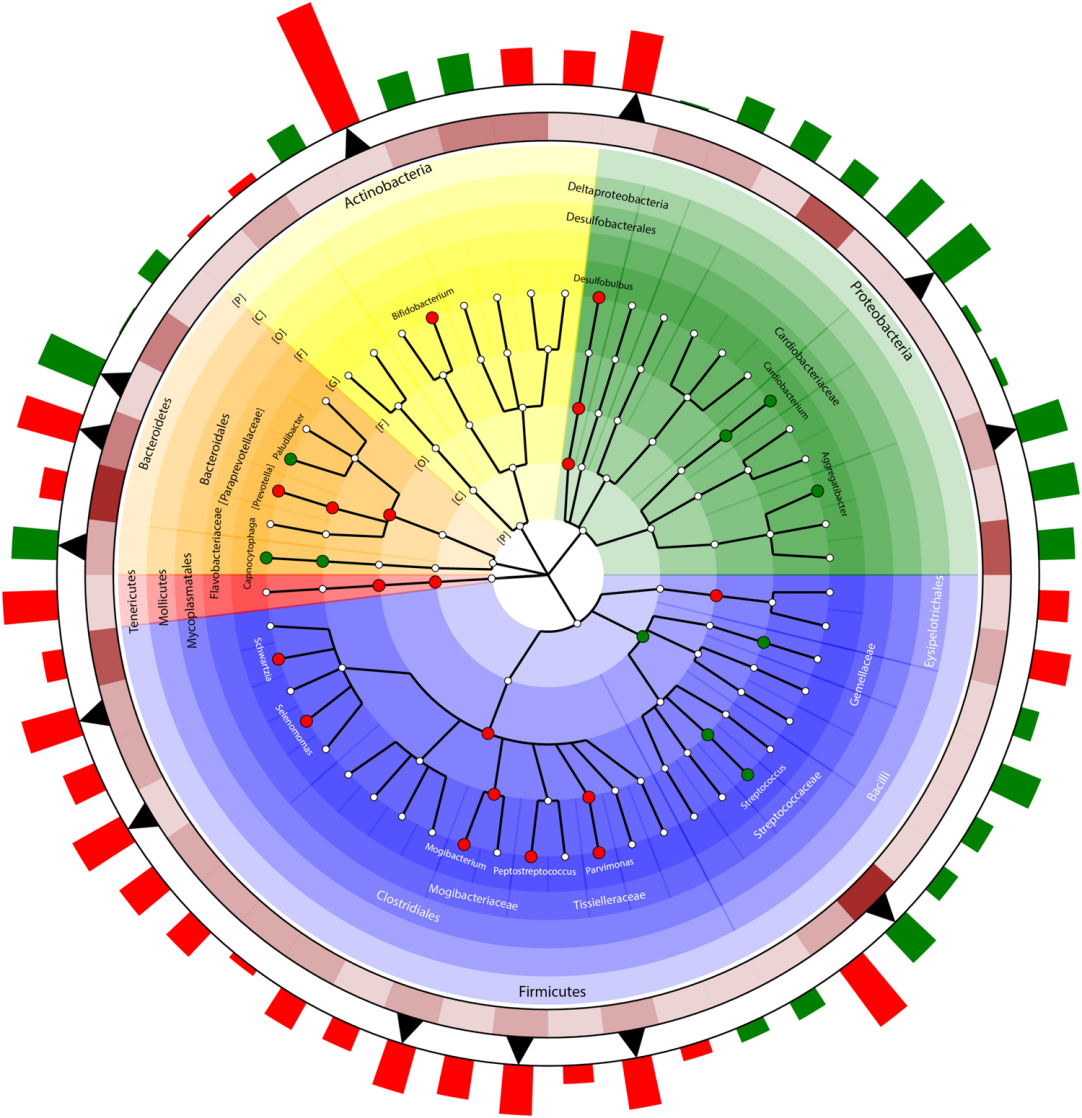
Cladogram representing the taxa associated with greater incense burning exposure. The cladogram shows the classification-based relationships of selected taxa involved in the study (Phylum [P], class [C], order [O], family [F] and genus [G] levels). Clades are colored by phylum. Only taxa that were identified in the trend analysis by DESeq. 2 with statistical significance (q < 0.1, i.e., after FDR correction) are reported. Red and green nodes indicate enrichment and depletion, respectively, for that particular taxon as exposure to incense increases. The first inner ring represents a heatmap showing mean abundance of the genera adjusted for size factors in DESeq2 (>0.1%; 0.1–0.99%; 1–5%; 5–10%; >10%). Bars on the external ring represent log2 fold changes of taxa mean counts per unit of increased incense exposure at the genus level. Red bars indicate enrichment of a genus while green bars indicate depletion, as detected by the DESeq2 analysis. Triangles underneath the log2 fold change bars indicate statistical significance with q < 0.1. The cladogram was built using Graphlan^53^.

We investigated whether increased incense exposure was associated with differences in the relative abundance of specific bacterial taxa in the oral microbial community using negative binomial generalized linear models, as implemented by the DESeq function in the DESeq2 R package^20^ (Fig. 2, Table 2, Supplementary Tables S2 and S3) adjusting for age, gender, smoking habits and assay batch. Trends of depletion and enrichment, with respect to incense use, tended to follow taxonomic affiliations within the phyla, particularly for *Firmicutes* (within the class *Clostridia*, 15 out of 16 taxa (94%) were enriched, and within *Bacilli*, 8 out of 9 (88%) were depleted) and *Proteobacteria* (all taxa but one (92%) were depleted) (Table 2, Fig. 2, Supplementary Table S2).

**Table 2:** Selected differentially abundant taxa of the oral microbiome as exposure to incense increases.

Taxaa	Trend*				Never	Occasionall^d^		Frequentl^e^		Daily^f^	
	Mean	log2FC (CI 95%)	p	q^c^	Mean^b^	Mean^b^	log2FC (CI 95%)	Mean^b^	log2FC (CI 95%)	Mean^b^	log2FC (CI 95%)
**Phylum; Class**											
Firmicutes; Bacilli	18098.32	−0.12 (−0.21, −0.03)	0.01	0.07	23442.42	18623.02	−0.20 (−0.56, 0.16)	18092.38	−0.32 (−0.67, 0.04)	16759.61	−0.44 (−0.79,−0.08)
Proteobacteria; Deltaproteobacteria	0.90	0.12 (0.02, 0.22)	0.02	0.09	0.62	0.5	−0.24 (−0.73, 0.25)	0.67	−0.09 (−0.58, 0.40)	1.44	0.49 (0.00, 0.98)
Tenericutes; Mollicutes	26.83	0.17 (0.05, 0.29)	0.01	0.07	10.83	24.48	0.43 (−0.10, 0.96)	25.5	0.51 (−0.01, 1.02)	32.63	0.81 (0.30, 1.33)
**Phylum; Class; Order**											
Bacteroidetes; Bacteroidia; Bacteroidales	8050.98	0.12 (0.04, 0.21)	0.01	0.09	5603.61	7529.91	0.37 (0.01, 0.72)	8476.91	0.54 (0.20, 0.89)	8454.13	0.55 (0.20, 0.89)
Firmicutes; Clostridia; Clostridiales	4329.04	0.11 (0.03, 0.19)	0.01	0.09	4214.89	3861.05	−0.13 (−0.46, 0.21)	4073.68	−0.05 (−0.37, 0.27)	4907.67	0.21 (−0.12, 0.54)
Firmicutes; Erysipelotrichi; Erysipelotrichales	72.86	0.13 (0.02, 0.25)	0.02	9.70E-02	36.98	66.52	0.63 (0.16, 1.11)	87.35	1.02 (0.56, 1.48)	70.11	0.76 (0.30, 1.22)
Proteobacteria; Deltaproteobacteria; Desulfobacterales	0.93	0.14 (0.03, 0.26)	0.01	0.09	0.61	0.52	−0.23 (−0.89, 0.33)	0.72	−0.05 (−0.61, 0.52)	1.48	0.57 (0.01, 1.13)
Tenericutes; Mollicutes; Mycoplasmatales	18.81	0.16 (0.03, 0.29)	0.02	0.09	4.14	17.78	0.73 (0.13, 1.32)	16.19	0.61 (0.02, 1.19)	24.69	1.03 (0.44, 1.61)
**Phylum; Class; Order; Family**											
Bacteroidetes; Bacteroidia; Bacteroidales; [Paraprevotellaceae]	769.04	0.20 (0.08, 0.31)	7.26E-04	0.03	401.05	681.84	0.57 (0.09, 1.04)	786.63	0.75 (0.29, 1.21)	879.52	0.89 (0.43, 1.35)
Bacteroidetes; Flavobacteriia; Flavobacteriales; Flavobacteriaceae	147.53	−0.15 (−0.26, −0.04)	0.01	0.07	201.21	163.74	−0.15 (−0.62, 0.32)	144.35	−0.44 (−0.90, 0.01)	129.64	−0.49 (−0.95,−0.03)
Firmicutes; Bacilli; Gemellaceae	1149.33	−0.14 (−0.24, −0.04)	0.01	0.07	1433.1	1231.63	−0.03 (−0.45, 0.39)	1209.3	−0.16 (−0.56, 0.24)	984.51	−0.38 (−0.79, 0.02)
Firmicutes; Bacilli; Lactobacillales; Streptococcaceae	14602.46	−0.13 (−0.22, −0.04)	3.21E-03	0.06	19687.79	15511.37	−0.23 (−0.60, 0.13)	13881.31	−0.43 (−0.78,−0.08)	13727.46	−0.48 (−0.83,−0.12)
Firmicutes; Clostridia; Clostridiales; [Mogibacteriaceae]	118.31	0.14 (0.03, 0.25)	0.01	0.07	68.15	114.2	0.53 (0.09, 0.98)	122.73	0.65 (0.22, 1.08)	126.19	0.70 (0.27, 1.14)
Firmicutes; Clostridia; Clostridiales; [Tissierellaceae]	108.59	0.15 (0.03, 0.27)	0.02	0.09	56.51	104.67	0.57 (0.05, 1.08)	112.83	0.70 (0.20, 1.21)	116.88	0.78 (0.27, 1.28)
Proteobacteria; Gammaproteobacteria; Cardiobacteriales; Cardiobacteriaceae	20.94	−0.18 (−0.31, −0.04)	0.01	0.07	34.11	21.69	−0.54 (−1.10, 0.02)	19.63	−0.82 (−1.37,−0.28)	19.23	−0.79 (−1.33,−0.24)
**Phylum; Class; Order; Family; Genus**											
Actinobacteria; Actinobacteria; Bifidobacteriales; Bifidobacteriaceae; Bifidobacterium	11.14	0.46 (0.27, 0.64)	1.15E-06	5.88E-05	4.28	4.1	−0.61 (−1.31, 0.08)	7.94	0.14 (−0.55, 0.82)	20.19	1.14 (0.46, 1.81)
Bacteroidetes; Bacteroidia; Bacteroidales; [Paraprevotellaceae]; [Prevotella]	737.22	0.21 (0.09, 0.34)	7.38E-04	0.02	428.37	640.04	0.45 (−0.03, 0.93)	755.97	0.67 (0.21, 1.14)	842.4	0.81 (0.34, 1.28)
Bacteroidetes; Bacteroidia; Bacteroidales; Porphyromonadaceae; Paludibacter	24.48	−0.22 (−0.38, −0.06)	0.01	0.06	54.57	22.66	−0.95 (−1.56,−0.34)	22.53	−0.94 (−1.54,−0.35)	21.97	−1.04 (−1.63,−0.44)
Bacteroidetes; Flavobacteriia; Flavobacteriales; Flavobacteriaceae; Capnocytophaga	147.68	−0.16 (−0.29, −0.04)	0.01	0.07	205.51	157.82	−0.21 (−0.70, 0.28)	149.64	−0.44 (−0.91, 0.04)	128.27	−0.53 (−1.01,−0.05)
Firmicutes; Bacilli; Lactobacillales; Streptococcaceae; Streptococcus	14718.21	−0.15 (−0.25, −0.05)	4.10E-03	0.05	19472.46	15351.19	−0.24 (−0.64, 0.15)	14943.98	−0.32 (−0.71, 0.06)	13196.7	−0.51 (−0.90,−0.13)
Firmicutes; Clostridia; Clostridiales; [Mogibacteriaceae]; Mogibacterium	37.92	0.17 (0.05, 0.29)	0.01	0.05	21.28	35.45	0.60 (0.14, 1.07)	39.23	0.74 (0.29, 1.19)	41.43	0.81 (0.36, 1.26)
Firmicutes; Clostridia; Clostridiales; [Tissierellaceae]; Parvimonas	101.51	0.18 (0.04, 0.31)	0.01	0.07	53.14	94.09	0.58 (0.05, 1.10)	108.32	0.78 (0.27, 1.29)	109.04	0.81 (0.29, 1.32)
Firmicutes; Clostridia; Clostridiales; Peptostreptococcaceae; Peptostreptococcus	87.22	0.18 (0.03, 0.33)	0.02	0.08	40.78	74.41	0.68 (0.10, 1.26)	110.12	1.17 (0.61, 1.63)	82.85	0.88 (0.31, 1.44)
Firmicutes; Clostridia; Clostridiales; Veillonellaceae; Schwartzia	7.78	0.19 (0.04, 0.35)	0.02	0.08	6.7	6.21	−0.08 (−0.69, 0.53)	7.28	0.11 (−0.48, 0.70)	9.51	0.47 (−0.13, 1.06)
Firmicutes; Clostridia; Clostridiales; Veillonellaceae; Selenomonas	136.12	0.20 (0.07, 0.33)	3.30E-03	0.05	127.28	99.69	−0.36 (−0.87, 0.14)	133.15	−0.03 (−0.52, 0.47)	165.18	0.29 (−0.20, 0.79)
Proteobacteria; Deltaproteobacteria; Desulfobacterales; Desulfobulbaceae; Desulfobulbus	0.86	0.21 (0.03, 0.39)	0.02	0.08	0.47	0.53	−0.18 (−0.85, 0.50)	0.64	−0.02 (−0.70, 0.65)	1.36	0.64 (−0.03, 1.31)
Proteobacteria; Gammaproteobacteria; Cardiobacteriales; Cardiobacteriaceae; Cardiobacterium	20.86	−0.21 (−0.36, −0.06)	0.01	0.06	35.46	20.68	−0.65 (−1.24,−0.07)	20.64	−0.88 (−1.45,−0.32)	18.47	−0.89 (−1.46,−0.32)
Proteobacteria; Gammaproteobacteria; Pasteurellales; Pasteurellaceae; Aggregatibacter	332.36	−0.17 (−0.31, −0.03)	0.02	0.08	418.86	397.33	0.02 (−0.53, 0.57)	311.36	−0.40 (−0.93, 0.13)	292.27	−0.42 (−0.96, 0.11)

^a^Only those taxa that had a significantly differential abundance with q < 0.1 and a Cook’s distance < 10 for the trend analysis are shown.

^b^Mean values refer to mean normalized counts of taxa according to incense burning group.

^c^FDR adjusted p value implemented independently at each level (i.e. phylum, class …).

^d^Occasional users report to burn incense in the household one time or less a week.

^e^Frequent users report to burn incense in the household 2–5 times a week.

^f^Daily users report to burn incense in the household 5–7 times a week.

*Trend analysis corresponds to the log2 fold change per unit of change of the continuous-valued incense variable.

Greater incense use was associated with depletion of the *Firmicutes* class *Bacilli*, largely due to depletion of the high-abundance genus *Streptococcus*, while *Firmicutes* order *Clostridiales* exhibited increased abundance, related partially to a number of minor genera (*Schwartzia*, *Mogibacterium*, *Parvimonas*, *Peptostreptococcus* and *Selenomonas*) (Table 2, Fig. 2, Supplementary Table S2). Greater incense use was associated with increased abundance of *Bacteroidetes* order *Bacteroidales* and its genus [*Prevotella*], while the *Bacteroidales* genus *Paludibacter* and order *Flavobacteriales* genus *Capnocytophaga* exhibited decreased abundance. Among *Proteobacteria*, genera *Aggregatibacter* and *Cardiobacterium* exhibited depletion; only the genus *Bifidobacterium* in *Actinobacteria* was identified as significantly enriched as exposure to incense increased. The same trend analysis across incense groups was independently performed for nonsmokers (n = 190) and smokers (n = 83); the direction of association between incense use and oral bacterial abundance was largely replicated in both of these groups (e.g., *Streptococcus*, *Clostridiales*, [*Prevotella*]), although some inconsistent associations between these two groups were observed (e.g., *Mogibacterium*, *Peptostreptococcus* and *Cardiobacterium*) (Supplementary Table S3). A variance partitioning analysis^21^ attributed 0.7% of the variance to incense use, and 0.6% to smoking, with only 0.09% shared by both variables. These two independent variance components were highly statistically significant (p ≤ 0.005). We also found that even occasional incense use (once a week or less) tended to be associated with taxon abundance differentials (never vs. occasional, Supplementary Table S4).

### Correlation networks amongst selected taxa

**Figure 3:**
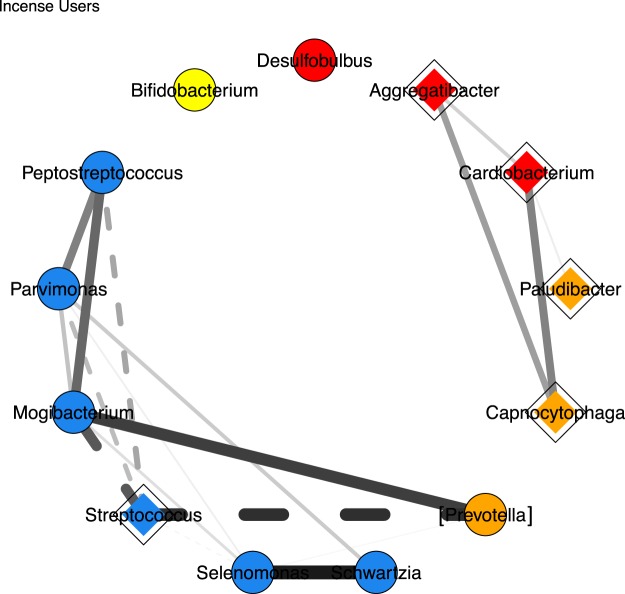
Correlation network showing interactions amongst selected genera in incense users. Only those genera that were identified as being significantly associated to increased levels of incense are displayed. Colors represent phylum affiliation (Actinobacteria: yellow; Bacteroidetes: orange; Firmicutes: blue; Proteobacteria: red). Diamonds indicate depletion and circles indicate enrichment of taxa as exposure to incense increases. The thickness of the lines designates the strength of the correlation. Solid lines indicate positive correlations and dashed lines indicate negative correlations. Only those correlations ≥0.3 or ≤−0.3 are shown. Correlations were calculated using DESeq. 2-normalized abundance and the Spearman’s rank correlation coefficient, and significance was computed and adjusted using the FDR correction for multiple testing^50^.

We built a correlation network with all incense users by means of the nonparametric Spearman correlations coefficient with the thirteen genera that were identified as being significantly associated with incense exposure in the trend analysis (Table 2, Fig. 3). A correlation network allows to detect whether changes in taxa abundances are occurring in the same direction or not. Two subcomponents in the correlation network were observed. The first one comprised most of the depleted taxa, *Aggregatibacter*, *Cardiobacterium*, *Capnocytophaga*, *Paludibacter*, all of which are positively correlated. The second and larger one comprised six of the eight enriched taxa and *Streptococcus* (depleted with incense use). The latter subcomponent comprised all six *Firmicutes* identified as associated with incense exposure and the *Bacteroidetes* [*Prevotella*]. *Streptococcus* was negatively associated with most of the other members of this network subcomponent. No significant correlations involving the taxa *Bifidobacterium* and *Desulfobulbus* were observed.

## Discussion

This study explored for the first time the potential effect that household incense use has on the oral microbiota. We found that incense use was associated with increased diversity and changes in overall structure and composition of the oral microbial community. As the frequency of incense use increased, several taxa tended toward lower abundance, including *Streptococcus*, the dominant genus of the oral microbiota, and *Paludibacter*, *Capnocytophaga*, *Aggregatibacter* and *Cardiobacterium*, while others tended toward increased abundance, including *Bacteroidales*, and its genus [*Prevotella*], and the *Actinobacteria* genus *Bifidobacterium*. Overall, bacterial abundances were altered with increasing levels of incense use, but notably abundance differentials were observed even in occasional users (once a week or less), suggesting that incense use impacts the oral microbiota even at relatively low levels of exposure.

Incense burning has a long history in many cultures and is commonly used for religious ceremonies, aromatizing and other reasons. In the Arabian gulf, and the UAE in particular, incense is frequently burned in households, but also in mosques and shopping malls, being one of the most common sources of indoor smoke exposure^4,22^. Incense burning produces fine and ultrafine airborne particulates in large quantities when compared to other indoor air pollutant sources (i.e. cooking) and emits gaseous pollutants, including carbon monoxide (CO), sulfur dioxide (SO_2_), nitric oxide (NO) and volatile organic compounds (VOCs) in relatively high concentrations^1,8^, as well as other aromatic, irritant and toxic compounds^10^.

Incense smoke presents many similarities in composition to tobacco smoke. Hence, exposure to incense could potentially have similar effects on the oral environment and on the function and composition of saliva, as observed in tobacco smokers^23,24^. Thus, we could expect that incense use, as with tobacco use, could result in the reduction of saliva by the depletion of oxygen^25^, increased acidity^26,27^, decreased activity and abundances of salivary proteins^28,29^, and lower abundance of Immunoglobulin A (IgA) molecules^30^. We recently reported^19^ that cigarette use in this population is strongly related to *Proteobacteria* and *Fusobacteria* depletion and enrichment of several taxa within the phyla *Synergistetes* and *Actinobacteria*, among others^19^. Despite incense and tobacco smoke potentially having similar effects on the oral environment, our results indicate that the effect of incense exposure on microbiota composition differ from those of tobacco exposure. First, the impacted bacteria are largely dissimilar from those associated with tobacco use and second, the microbial differentials observed with incense use are similar whether an individual used tobacco products or not. In fact, our variance partitioning showed that the effects of incense use and smoking on the oral microbiota are largely independent. This could in part be due to toxicants present in incense use that are absent in tobacco smoke and that might have differential cytotoxic or mutagenic effects on the bacterial community^10^, potentially affecting the oral environment in distinctive ways.

One of the most striking results of our study was the observed depletion of the most abundant taxa of the oral microbiota, the *Streptococci*. These are, for the most part, commensal bacteria with high ability to adhere to hard and mucosal oral surfaces, as most possess a diverse array of adhesins^31^. If incense use decreases the activity and abundance of salivary proteins^28,29^, this may compromise the ability of Streptococci to efficiently adhere and colonize oral surfaces and instead, facilitate their agglutination and removal by swallowing^31^. In addition to enabling bacterial adherence, proteins present in the saliva are also an important source of nutrients. When salivary proteins become a limiting factor, highly proteolytic bacteria will outcompete low proteolytic bacteria^32^. This is in agreement with the patterns observed (Table 2, Supplementary Table S2), as depleted *Streptococci* have a low proteolytic potential^33^, while *Bifidobacterium* (*Actinobacteria*), *Parvimonas* and *Peptostreptococcus* (*Firmicutes*; *Clostridia*), which comprise highly proteolytic species^34–36^ were enriched.

Bacteria are organisms that interact within and between species forming complex communities that influence the niche they inhabit and that can respond to external stimuli as well^37^. The interactions between members of a bacterial community are for the most part non-random and thus, shape the structure and composition of that particular community^38^. While synergistic interactions between members of the community will stimulate the growth of one another, antagonist effects will lead to the depletion of one or more of the organisms^32^. For example, a synergistic interaction is known for *Prevotella*, as it interacts with *Peptostreptococcus* by providing amino acids to the medium which are used by *Peptostreptococcus* promoting its growth^39^. Similarly, *Schwartzia* is a bacterium limited by being able to metabolize only succinate^40^, but its association with a succinate producer such as *Selenomonas* will ensure that the growth of one will enable the growth of the other^40^. Both of the latter associations (*Prevotella*-*Peptostreptococcus* and *Schwartzia*-*Selenomomas*) were observed as positive correlations in the network analysis (Fig. 3). Nevertheless, further research is needed, as positive correlations could also mean that both taxa respond in a similar way to a perturbation or that they consume similar resources but are in different niches of the oral cavity.

This study is the first to our knowledge that investigates the association between use of incense and the oral microbiome. We recognize that the amount of variance explained by incense was not large, however, this likely reflects the large individual differences in overall microbiome composition among the participants in our study. The use of the structured questionnaire was important to reveal the extent to which different frequencies of incense burning in the household where associated to oral microbiome dysbiosis. Our study had some limitations such as (1) the low number of nonusers of incense, since household incense use is traditional in UAE households, (2) the absence of data related to dental history and dental hygiene habits, an important factor influencing the presence of particular non-pathogenic and pathogenic bacteria, (3) the lack of information regarding the type of incense used and (4) the low number of participants that smoke but did not burn incense (only two), preventing the comparison between exclusively incense users and exclusively smokers. Also, we were unable to track the progression of microbiota changes with continued incense use, as only one oral sample was collected per individual. Finally, although *16S rRNA* sequencing provides information on the bacterial composition and abundances of the oral taxa, information on functional potential of these microbes can only indirectly be inferred from these data. The latter will be assessed in future studies.

In summary, our results demonstrate that indoor incense use is associated with oral microbiota structure and composition, even with relatively infrequent use (as observed in occasional users). Furthermore, this association is largely independent of tobacco-related microbial perturbations (as no significant differences were observed between nonsmoker and smoker incense users). As the oral microbiome serves important functions in health^12,13^, the observed impact on oral microbiota may serve as an early biomarker of incense-related toxicities and related health consequences. Although an important indoor air pollutant, guidelines for control of incense use have yet to be developed.

## Materials and Methods

### Study subjects

This study used mouthwash samples and metadata from 303 subjects recruited by the UAEHFS-pilot study undertaken between the months of December 2014 and April 2015^41^. Study subjects were eligible to participate if they were Emirati nationals aged 18 and above. Participants completed physical and clinical exams, including measurements of body composition and blood pressure, provided blood, urine and mouthwash samples and completed a self-administered questionnaire including information on socio-demographic factors, lifestyle and medical history^41^. From 517 consented study subjects, 303 subjects completed baseline questionnaires including incense data and provided mouthwash samples (Supplementary Fig. S1). This study was approved by the Institutional Review Boards (IRB) of Sheikh Khalifa Medical City (SKMC), Zayed Military Hospital (ZMH), New York University Abu Dhabi (NYUAD) and NYU Langone Health, New York. All individuals participating in the study read and signed an informed consent. All experiments were performed in accordance with relevant guidelines and regulations.

### Measurements

#### Incense burning classification of study subjects

Incense burning habits were ascertained through a self-reported structured questionnaire and were classified as follow; Never users, occasional users (usually one or less times a week), frequent users (2 to 5 times a week) and daily users (5 to 7 times a week). (Supplementary Metadata).

#### Definition of smoker and nonsmoker

Smoker and nonsmoker status was defined as in^19^. Briefly, study subjects provided detailed information on smoking habits in a structured questionnaire. In addition, study subjects provided urine samples that were used to test for presence of cotinine, a nicotine metabolite, using the COT rapid test cassette (International Biomedical Supplies), with a cut off concentration of 200 ng/ml. Smokers were defined as all study subjects that self-reported as smoker independently of cotinine results. Nonsmokers were those study subjects that self-reported as nonsmokers and were further validated by a cotinine negative result.

#### Mouthwash sample collection

As reported previously^19^ study subjects were provided with 10 ml of pharmaceutical grade normal saline (0.9%) solution and asked to vigorously swish for 30 seconds and spit it out into a collection tube. Samples were initially stored at 4 °C. Once in the lab, samples were vortexed for 20 seconds, pipetted up and down and aliquoted into 1 ml cryotubes to be stored at −80 °C until further processing. DNA extractions were performed on two blank saline samples alongside the mouthwash samples to confirm that the saline solution used for collection of the mouthwash samples contained no detectable levels of DNA. DNA concentrations were quantified using the high sensitivity Qubit assay. Only mouthwash samples yielded measurable amounts of DNA^19^.

#### Microbiome assay

Briefly, 2 ml were used per mouthwash sample for DNA extraction. Cell pellets were collected by spinning samples at 6000 g for 3 min and then at 10000 g for 10 min. DNA was extracted using the Mo BioPowerSoil PowerLyzer kit following manufacturer’s instructions (Mo Bio Laboratory Inc, California, USA). Genomic DNA was visualized on a gel and quantified using the Qubit HS kit (Thermo Fisher Scientific). Amplification of DNA from the V4 region of *16S rRNA* gene (515F -5′GTGCCAGCMGCCGCGGTAA3′ - 806R - 5′GGACTACHVGGGTWTCTAAT3′) was performed using specifically designed primers with Illumina adaptor sequences and a 12 bp index (reverse primer only) added for posterior multiplexing. The FastStart enzyme (Roche, IN) was used to amplify the *16S rRNA gene* for 27 cycles using approximately 12.5 ng of DNA per sample. PCR products were visualized in an agarose gel, purified using Agencourt AMPure beads (Beckman Coulter Life Sciences, IN) and quantified using the Qubit BR kit (Thermo Fisher Scientific). Samples were then pooled for sequencing on an Illumina MiSeq platform in two batches^42^.

#### Quality control

As previously reported, samples were sequenced in two batches^42^. In addition, three samples were triplicated in each batch to assess sample quality control. A blank was used in each batch for both DNA extractions and PCR amplifications. DNA concentrations were measured each time to verify that blanks were devoid of significant amounts of DNA. Only one of them was sequenced with each sequencing batch. Quality control samples showed good consistency, with the coefficient of variability ranging from 1.65–2.32% for the Shannon entropy and 1.02–7.21% for specific phyla relative abundances^19^.

### Statistical analysis

#### Sequence processing and taxonomic assignment

The QIIME 1 bioinformatic pipeline was used to process the sequence data. Sequences were de-multiplexed and trimmed using the split_libraries_fastq.py QIIME script^43^. Poor quality sequences were excluded from further analyses (minimum average base score quality per read was 20, minimum read length 200 bp and no mismatches in adaptor or barcode sequences was permitted). The pick_de_novo_otus.py workflow as implemented in QIIME^43^ to cluster sequences into operational taxonomical units (OTU) using a 97% pairwise-identity cutoff. UCLUST^44^, PyNAST^45^ and the Greengenes database were further implemented and used respectively to obtain taxonomical assignment of the sequences. Removal of chimeric sequences was accomplished using ChimeraSlayer as implemented in the QIIME workflow^46^. Low count OTUs were filtered from the analyses if they were singletons and absent in more than 10% of the samples.

#### Alpha diversity

Richness and Shannon entropy were estimated to assess the within-sample diversity. Both indexes were estimated for 200 iterations of rarefied OTU datasets (16738 sequences per sample) followed by computing the average for each sample using the vegan library in R^47^. Multiple linear regression adjusting for age, gender, smoking status and batch was implemented to compare alpha-diversity scores between different incense burning groups (never, occasionally, frequently and daily).

#### Microbial composition according to incense burning

We examined the relationship between oral microbiome composition and incense burning by conducting a permutational multivariate analysis of variance (PERMANOVA) of weighted (taxa relative abundances) and unweighted (binary input, absence/presence) UniFrac distance matrices^47^. The UniFrac distance metric incorporates phylogenetic distances between community organisms into the calculations of the dissimilarity matrices and were computed using the UniFrac function in the Phyloseq library in R^48,49^. The latter was visualized through Principal Coordinate Analyses (PCoA) after correction for negative eigenvalues. All PERMANOVA analyses were adjusted for age, gender, smoking status and sequencing batch, and were performed using the Adonis function in the vegan R library^47^.

#### Identification of differences in taxa abundances as exposure to incense burning increases

We studied differences of taxa relative abundances associated to increasing incense exposure using a model based on negative binomial distribution as implemented by the DESeq function in the DESeq2 R package^20^. The trend analysis was executed by using the continuous-valued incense variable. Since the average use of incense by the participants was not reported in the questionnaire, value 1 was attributed to “Never”, 2 to “Occasionally”, 3 to “Frequently, and 4 to “Daily” users. Log_2_ fold change results reported for the trend analysis correspond to the log_2_ fold change per unit of change of the continuous-valued incense variable. Comparisons between incense frequency categories were done by including in the model the contrast argument and specifying the comparison of interest. A p-value < 0.05 was considered of nominal statistical significance, and an FDR-adjusted^50^ p-value < 0.10 (named hereafter q-value) was considered significant after adjustment for multiple comparisons. Analyses were done at all taxonomical levels and models were adjusted for age, gender, smoking status and sequencing batch. All analyses were conducted using R version 3.3.2^51^. Where relevant, we assessed the separate contributions of different factors to the variance in microbiota composition using a variance partitioning analysis^21^.

#### Correlation networks of selected taxa

We investigated potential interactions between genera by network analysis of taxa co-occurrence patterns according to frequency of exposure to incense burning. Correlations were calculated using the Spearman’s rank correlation coefficient and significance was computed and adjusted using the FDR correction for multiple testing^50^. Only those correlations ≥0.3 or ≤−0.3 are shown. This analysis was performed and visualized using the qgraph package in R^52^.

## Supplementary information

Supplementary information

Supplementary Metadata

## Data Availability

The dataset (BIOM file) used and analyzed during the current study is available in the Qiita database study ID – 11838 and the metadata is provided as Supplementary Metadata.

## References

[1] Lin TC, Krishnaswamy G, Chi DS (2008). Incense smoke: clinical, structural and molecular effects on airway disease. Clin Mol Allergy.

[2] Pan A (2014). Incense use and cardiovascular mortality among Chinese in Singapore: the Singapore Chinese Health Study. Environ Health Perspect.

[3] Cohen R, Sexton KG, Yeatts KB (2013). Hazard assessment of United Arab Emirates (UAE) incense smoke. Sci Total Environ.

[4] Yeatts KB (2012). Indoor air pollutants and health in the United Arab Emirates. Environ Health Perspect.

[5] Lee WH, Choo JY, Son JY, Kim H (2016). Association between long-term exposure to air pollutants and prevalence of cardiovascular disease in 108 South Korean communities in 2008–2010: A cross-sectional study. Sci Total Environ.

[6] Franklin BA, Brook R, Arden Pope C (2015). Air pollution and cardiovascular disease. Curr Probl Cardiol.

[7] Tse LA, Yu IT, Qiu H, Au JS, Wang XR (2011). A case-referent study of lung cancer and incense smoke, smoking, and residential radon in Chinese men. Environ Health Perspect.

[8] Jetter JJ, Guo Z, McBrian JA, Flynn MR (2002). Characterization of emissions from burning incense. Sci Total Environ.

[9] Chen CC, Lee H (1996). Genotoxicity and DNA adduct formation of incense smoke condensates: comparison with environmental tobacco smoke condensates. Mutat Res.

[10] Zhou R (2015). Higher cytotoxicity and genotoxicity of burning incense than cigarette. Environ Chem Lett.

[11] Rodgman, A. & Perfetti, T. *The Chemical Components of Tobacco and Tobacco Smoke*. (CRC Press, Taylor & Francis Group, 2009).

[12] Wilson, M. *Microbial inhabitants of humans: their ecology and role in health and disease*. (Cambridge University Press, 2005).

[13] Human Microbiome Project, C (2012). Structure, function and diversity of the healthy human microbiome. Nature.

[14] Zaura E, Nicu EA, Krom BP, Keijser BJ (2014). Acquiring and maintaining a normal oral microbiome: current perspective. Front Cell Infect Microbiol.

[15] Wu J (2016). Cigarette smoking and the oral microbiome in a large study of American adults. Isme J.

[16] Fan, X. *et al*. Drinking alcohol is associated with variation in the human oral microbiome in a large study of American adults. (in review). 10.1186/s40168-018-0448-x PMC5914044 29685174

[17] Yu G (2017). The effect of cigarette smoking on the oral and nasal microbiota. Microbiome.

[18] Camelo-Castillo AJ (2015). Subgingival microbiota in health compared to periodontitis and the influence of smoking. Front Microbiol.

[19] Valles, Y., Inman, BA, P. & Ahn, J. Types of tobacco consumption and the oral microbiome in the United Arab Emirates Healthy Future (UAEHFS) Pilot Study. *Scientific Reports* (2018). 10.1038/s41598-018-29730-x PMC6063860 30054546

[20] Love MI, Huber W, Anders S (2014). Moderated estimation of fold change and dispersion for RNA-seq data with DESeq. 2. Genome Biol.

[21] Borcard D, Legendre P, Drapeau P (1992). Partialling out the Spatial Component of Ecological Variation. Ecology.

[22] Abdul Wahab A, Mostafa OA (2007). Arabian incense exposure among Qatari asthmatic children. A possible risk factor. Saudi Med J.

[23] Nagler R (2000). Effect of cigarette smoke on salivary proteins and enzyme activities. Arch Biochem Biophys.

[24] Zuabi O (1999). The effect of smoking and periodontal treatment on salivary composition in patients with established periodontitis. J Periodontol.

[25] Kenney EB, Saxe SR, Bowles RD (1975). The effect of cigarette smoking on anaerobiosis in the oral cavity. J Periodontol.

[26] Kanwar AJ, Mahajan R, Parsad D (2013). Effect of age at onset on disease characteristics in vitiligo. J Cutan Med Surg.

[27] Parvinen T (1984). Stimulated salivary flow rate, pH and lactobacillus and yeast concentrations in persons with different types of dentition. Scand J Dent Res.

[28] Granger DA (2007). Individual differences in salivary cortisol and alpha-amylase in mothers and their infants: relation to tobacco smoke exposure. Dev Psychobiol.

[29] Taniguchi M (2013). Multimolecular salivary mucin complex is altered in saliva of cigarette smokers: detection of disulfide bridges by Raman spectroscopy. Biomed Res Int.

[30] Shilpashree HS, Sarapur S (2012). Evaluation of salivary immunoglobulin A levels in tobacco smokers and patients with recurrent aphthous ulcers. J Nat Sci Biol Med.

[31] Jenkinson HF, Lamont RJ (1997). Streptococcal adhesion and colonization. Crit Rev Oral Biol Med.

[32] Kuramitsu HK, He X, Lux R, Anderson MH, Shi W (2007). Interspecies interactions within oral microbial communities. Microbiol Mol Biol Rev.

[33] Jakubovics NS, Yassin SA, Rickard AH (2014). Community interactions of oral streptococci. Adv Appl Microbiol.

[34] Abu-Taraboush HM, al-Dagal MM, al-Royli MA (1998). Growth, viability, and proteolytic activity of bifidobacteria in whole camel milk. J Dairy Sci.

[35] Murdoch DA (1998). Gram-positive anaerobic cocci. Clin Microbiol Rev.

[36] Ota-Tsuzuki C, Alves Mayer MP (2010). Collagenase production and hemolytic activity related to 16S rRNA variability among Parvimonas micra oral isolates. Anaerobe.

[37] Stubbendieck RM, Vargas-Bautista C, Straight PD (2016). Bacterial Communities: Interactions to Scale. Front Microbiol.

[38] Prosser JI (2007). Essay - The role of ecological theory in microbial ecology. Nat Rev Microbiol.

[39] Pybus V, Onderdonk AB (1998). A commensal symbiosis between Prevotella bivia and Peptostreptococcus anaerobius involves amino acids: potential significance to the pathogenesis of bacterial vaginosis. FEMS Immunol Med Microbiol.

[40] Rainey, F. In *Bergey’s Manual of Systematic Bacteriology* Vol. Three (eds P De Vos *et al*.) Ch. The Firmicutes, 736–1190 (Springer, 2009).

[41] Abdulle A (2018). The UAE healthy future study: a pilot for a prospective cohort study of 20,000 United Arab Emirates nationals. BMC Public Health.

[42] Vallès Y (2018). Types of tobacco consumption and the oral microbiome in the United Arab Emirates Healthy Future (UAEHFS) Pilot Study. Sci Rep.

[43] Caporaso JG (2010). QIIME allows analysis of high-throughput community sequencing data. Nat Methods.

[44] Edgar RC (2010). Search and clustering orders of magnitude faster than BLAST. Bioinformatics.

[45] Caporaso JG (2010). PyNAST: a flexible tool for aligning sequences to a template alignment. Bioinformatics.

[46] Haas BJ (2011). Chimeric 16S rRNA sequence formation and detection in Sanger and 454-pyrosequenced PCR amplicons. Genome Res.

[47] Oksanen, J. *et al*. vegan: Community Ecology Package (2017).

[48] Lozupone CA, Hamady M, Kelley ST, Knight R (2007). Quantitative and qualitative beta diversity measures lead to different insights into factors that structure microbial communities. Appl Environ Microbiol.

[49] McMurdie PJ, Holmes S (2013). phyloseq: an R package for reproducible interactive analysis and graphics of microbiome census data. PLoS One.

[50] Benjamini Y, Hochberg Y (1995). Controlling the False Discovery Rate - a Practical and Powerful Approach to Multiple Testing. J Roy Stat Soc B Met.

[51] Team, R. C. R: A language and environment for statistical computing. R Foundation for Statistical Computing, Viena, Austria (2014).

[52] Epskamp S, Cramer AOJ, Waldorp LJ, Schmittmann VD, Borsboom D (2012). qgraph: Network Visualizations of Relationships in Psychometric Data. J Stat Softw.

[53] Asnicar F, Weingart G, Tickle TL, Huttenhower C, Segata N (2015). Compact graphical representation of phylogenetic data and metadata with GraPhlAn. PeerJ.

