# Supplementary material for: Incense Burning is Associated with Human Oral Microbiota Composition
Source: Sci Rep. 2019 Jul 11;9:10039. doi: 10.1038/s41598-019-46353-y (PMC6624419; doi:10.1038/s41598-019-46353-y)
Supplement: Supplementary file 1 — Supplementary information [file 41598_2019_46353_MOESM1_ESM.pdf]

Incense Burning Is Associated with Human Oral Microbiota Composition

Yvonne Vallès[1,2], Claire K. Inman[1], Brandilyn A. Peters[3,16], Laila Abdel Wareth[4], Abdishakur Abdulle[1], Habiba Alsafar[5,6], Fatme Al Anouti[7], Ayesha Al Dhaheri[8], Divya Galani[1], Muna Haji[1], Aisha Al Hamiz[1], Ayesha Al Hosani[1], Mohammed Al Houqani[9], Abdulla Aljunaibi[10], Marina Kazim[11], Tomas Kirchhoff[3,16], Wael Al Mahmeed[12], Fatma Al Maskari[13], Abdullah Alnaeemi[14], Naima Oumeziane[15], Ravichandran Ramasamy[16], Ann Marie Schmidt[16], Henri Vallès[2], Eiman Al Zaabi[11], Scott Sherman[1,3,17], Raghib Ali[1], Jiyoung Ahn[3,17] and Richard B. Hayes[3,17*]

[1]Public Health Research Center, New York University Abu Dhabi, Abu Dhabi, UAE;
[2]Department of Biological and Chemical Sciences, The University of the West Indies Cave Hill Campus, Cave Hill, Barbados;
[3]Department of Population Health, New York University School of Medicine, New York, USA;
[4]Pathology and Laboratory Medicine Institute, Cleveland Clinic, Abu Dhabi, UAE;
[5]Center for Biotechnology, Khalifa University of Science and Technology, Abu Dhabi, UAE;
[6]Biomedical Engineering Department, Khalifa University of Science and Technology, Abu Dhabi, UAE;
[7]College of Natural and Health Sciences, Zayed University, Abu Dhabi, UAE;
[8]Department of Nutrition, College of Food and Agriculture; UAE University, Al-Ain, UAE;
[9]Department of Medicine, College of Medicine and Health Sciences, UAE University, Al-Ain, UAE;
[10]Department of Pediatrics, Zayed Military Hospital, Abu Dhabi, UAE;
[11]Department of Pathology, Sheikh Khalifa Medical Center, Abu Dhabi, UAE;
[12]Heart and Vascular Institute, Cleveland Clinic, Abu Dhabi, UAE;
[13]Institute of Public Health, College of Medicine and Health Sciences, UAE University, Al-Ain, UAE.
[14]Department of Cardiology, Zayed Military Hospital, Abu Dhabi, UAE;
[15]Abu Dhabi Blood Bank, SEHA, Abu Dhabi, UAE;
[16]Diabetes Research Program, Division of Endocrinology, Diabetes and Metabolism, Department of Medicine, New York University School of Medicine, New York, USA;
[17]NYU Perlmutter Cancer Center, New York, USA

Supplementary Figure S1. Flow chart depicting the inclusion criteria of the participants involved in this study from the UAEHFS-pilot. UAEHFS-pilot participants were Emirati nationals aged 18 and above. Study participants completed a self-administered questionnaire including information on medical history. During the physical exam, participants provided blood, urine and mouthwash samples. From 517 consented study participants, 343 provided information on incense burning habits in the household and of these participants 303 gave an oral mouthwash sample. All individuals participating in the study read and signed an informed consent.

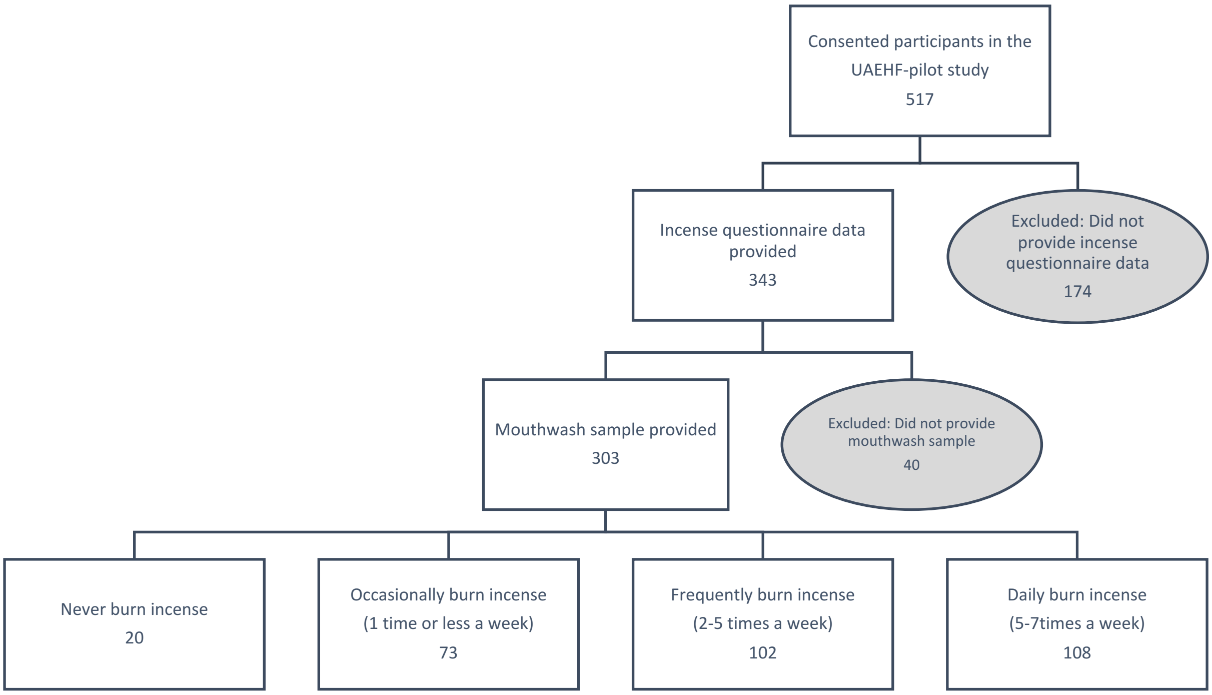

Supplementary Table S1. Summary table of the PERMANOVA showing the different factors originally included in the model. The interaction term between gender and incense use (Gender:Incense) was later removed as it was not statistically significant.

Variable	Df	SumsOfSqs	MeanSqs	F. Model	R2	Pr(>F)	
Age	1	0.2734	0.273382	4.3255	0.01378	0.001	***
Gender	1	0.0698	0.069820	1.1047	0.00352	0.327	
Batch	1	0.2842	0.284177	4.4963	0.01432	0.001	***
Nonsmoker	1	0.2411	0.241137	3.8153	0.01216	0.001	***
Incense	1	0.1985	0.198482	3.1404	0.01001	0.002	**
Gender:Incense	1	0.0629	0.062904	0.9953	0.00317	0.389	
Residuals	296	18.7080	0.063203		0.94304		
total	302	19.8379			1.00000		

Adonis model:
adonis(WeigthedUnifracMatrix~ Age + Gender + Nonsmoker + Batch + Gender*Incense + Incense, data)

Supplementary Table S2. Relative abundance trends of all the taxa observed in the oral microbiota as exposure to incense increases.

Taxa	Mean	Log2FC (95% CI)	FC (95% CI)	p	q[1]
Phylum					
Chloroflexi	0.42	0.04 (-0.08, 0.16)	1.03 (0.91, 1.15)	0.52	0.63
GN02	2.82	-0.09 (-0.23, 0.04)	0.94 (0.80, 1.07)	0.17	0.41
Tenericutes	25.34	0.18 (0.04, 0.31)	1.13 (0.99, 1.27)	0.01	0.14
SR1	70.87	0.10 (-0.04, 0.24)	1.07 (0.93, 1.21)	0.15	0.41
TM7	0.63	0.01 (-0.12, 0.15)	1.01 (0.87, 1.15)	0.85	0.93
Proteobacteria	6445.98	-0.06 (-0.16, 0.04)	0.96 (0.85, 1.06)	0.26	0.41
Spirochaetes	184.09	0.13 (0.00, 0.26)	1.09 (0.96, 1.22)	0.05	0.22
Synergistetes	23.82	0.07 (-0.06, 0.21)	1.05 (0.92, 1.19)	0.29	0.41
Firmicutes	21940.51	-0.05 (-0.14, 0.03)	0.96 (0.88, 1.05)	0.23	0.41
Actinobacteria	3039.92	0.002 (-0.11, 0.11)	1.00 (0.89, 1.11)	0.97	0.97
Cyanobacteria	4.46	-0.08 (-0.18, 0.01)	0.94 (0.85, 1.04)	0.09	NA
Fusobacteria	1670.85	0.04 (-0.04, 0.12)	1.03 (0.95, 1.11)	0.31	0.41
Bacteroidetes	8454.19	0.10 (0.00, 0.19)	1.07 (0.98, 1.16)	0.04	0.22
Phylum;Class					
Actinobacteria;Actinobacteria	2838.45	-0.04 (-0.14, 0.07)	0.98 (0.87, 1.08)	0.50	0.61
Actinobacteria;Coriobacteriia	186.83	0.01 (-0.11, 0.12)	1.00 (0.89, 1.12)	0.92	0.92
Bacteroidetes;Bacteroidia	8108.05	0.09 (0.01, 0.17)	1.07 (0.98, 1.15)	0.03	0.10
Bacteroidetes;Flavobacteriia	273.77	-0.10 (-0.20, 0.01)	0.94 (0.83, 1.04)	0.07	0.13
Chloroflexi;Anaerolineae	0.45	0.03 (-0.06, 0.13)	1.02 (0.93, 1.12)	0.51	0.61
Cyanobacteria;Chloroplast	5.33	-0.10 (-0.17, -0.03)	0.93 (0.86, 1.01)	0.01	NA
Firmicutes;Bacilli	18098.32	-0.12 (-0.21, -0.03)	0.92 (0.83, 1.01)	0.01	0.07
Firmicutes;Clostridia	4449.89	0.09 (0.01, 0.17)	1.06 (0.98, 1.14)	0.04	0.11
Firmicutes;Erysipelotrichi	71.93	0.11 (0.00, 0.21)	1.08 (0.97, 1.18)	0.05	0.12
Fusobacteria;Fusobacteriia	1699.38	0.03 (-0.04, 0.10)	1.02 (0.95, 1.09)	0.43	0.59
GN02;BD1-5	3.45	-0.10 (-0.21, 0.01)	0.94 (0.82, 1.05)	0.09	NA
Proteobacteria;Betaproteobacteria	2490.46	-0.08 (-0.19, 0.04)	0.95 (0.84, 1.06)	0.19	0.34
Proteobacteria;Deltaproteobacteria	0.90	0.12 (0.02, 0.22)	1.09 (0.99, 1.19)	0.02	0.09
Proteobacteria;Epsilonproteobacteria	162.38	-0.01 (-0.09, 0.06)	0.99 (0.92, 1.07)	0.73	0.77
Proteobacteria;Gammaproteobacteria	4013.86	-0.09 (-0.19, 0.00)	0.94 (0.84, 1.04)	0.06	0.13
Spirochaetes;Spirochaetes	201.37	0.13 (0.01, 0.25)	1.10 (0.98, 1.21)	0.03	0.10
Synergistetes;Synergistia	25.44	0.07 (-0.05, 0.19)	1.05 (0.93, 1.17)	0.25	0.41
Tenericutes;Mollicutes	26.83	0.17 (0.05, 0.29)	1.12 (1.00, 1.24)	0.01	0.07
Tenericutes;RF3	0.54	0.03 (-0.04, 0.10)	1.02 (0.95, 1.09)	0.43	0.59
TM7;TM7-3	0.67	0.02 (-0.09, 0.13)	1.02 (0.90, 1.13)	0.70	0.77
Phylum;Class;Order					
Actinobacteria;Actinobacteria;Actinomycetales	2613.68	0.00 (-0.10, 0.10)	1.00 (0.90, 1.10)	0.99	0.99
Actinobacteria;Actinobacteria;Bifidobacteriales	53.72	0.11 (-0.03, 0.24)	1.08 (0.94, 1.21)	0.11	0.25
Actinobacteria;Coriobacteriia;Coriobacteriales	183.65	0.03 (-0.09, 0.15)	1.02 (0.90, 1.14)	0.60	0.67
Bacteroidetes;Bacteroidia;Bacteroidales	8050.98	0.12 (0.04, 0.21)	1.09 (1.00, 1.18)	0.01	0.09
Bacteroidetes;Flavobacteriia;Flavobacteriales	262.54	-0.07 (-0.18, 0.03)	0.95 (0.85, 1.05)	0.16	0.30
Chloroflexi;Anaerolineae;Anaerolineales	0.46	0.04 (-0.07, 0.15)	1.03 (0.92, 1.14)	0.45	0.56
Cyanobacteria;Chloroplast;Streptophyta	5.19	-0.12 (-0.21, -0.04)	0.92 (0.83, 1.01)	0.01	NA
Firmicutes;Bacilli;Bacillales	6.12	-0.06 (-0.19, 0.06)	0.96 (0.83, 1.08)	0.32	0.45
Firmicutes;Bacilli;Gemellales	1148.02	-0.09 (-0.19, 0.01)	0.94 (0.84, 1.04)	0.06	0.16
Firmicutes;Bacilli;Lactobacillales	15389.85	-0.08 (-0.16, 0.01)	0.95 (0.86, 1.03)	0.07	0.16
Firmicutes;Clostridia;Clostridiales	4329.04	0.11 (0.03, 0.19)	1.08 (1.00, 1.16)	0.01	0.09
Firmicutes;Erysipelotrichi;Erysipelotrichales	72.86	0.13 (0.02, 0.25)	1.10 (0.98, 1.21)	0.02	0.10
Fusobacteria;Fusobacteriia;Fusobacteriales	1667.48	0.05 (-0.03, 0.12)	1.03 (0.96, 1.11)	0.20	0.33
Proteobacteria;Betaproteobacteria;Burkholderiales	179.82	-0.04 (-0.18, 0.09)	0.97 (0.84, 1.10)	0.50	NA
Proteobacteria;Betaproteobacteria;Neisseriales	2131.87	-0.05 (-0.17, 0.07)	0.97 (0.85, 1.09)	0.46	0.56
Proteobacteria;Betaproteobacteria;Thiobacterales	0.88	0.03 (-0.07, 0.13)	1.02 (0.92, 1.12)	0.57	NA
Proteobacteria;Deltaproteobacteria;Desulfobacterales	0.93	0.14 (0.03, 0.26)	1.10 (0.99, 1.22)	0.01	0.09
Proteobacteria;Epsilonproteobacteria;Campylobacterales	160.92	0.00 (-0.08, 0.08)	1.00 (0.92, 1.08)	0.92	0.96
Proteobacteria;Gammaproteobacteria;Cardiobacteriales	20.15	-0.12 (-0.24, 0.01)	0.92 (0.80, 1.05)	0.06	0.16
Proteobacteria;Gammaproteobacteria;Pasteurellales	3740.43	-0.07 (-0.17, 0.03)	0.95 (0.85, 1.05)	0.16	0.30
Proteobacteria;Gammaproteobacteria;Pseudomonadales	8.23	0.00 (-0.10, 0.10)	1.00 (0.90, 1.10)	0.93	NA
Spirochaetes;Spirochaetes;Spirochaetales	210.70	0.14 (0.01, 0.27)	1.10 (0.97, 1.23)	0.03	0.12
Synergistetes;Synergistia;Synergistales	26.14	0.08 (-0.05, 0.21)	1.06 (0.93, 1.19)	0.21	0.34
Tenericutes;Mollicutes;Mycoplasmatales	18.81	0.16 (0.03, 0.29)	1.12 (0.99, 1.25)	0.02	0.09
Tenericutes;Mollicutes;RF39	9.04	0.06 (-0.06, 0.19)	1.05 (0.92, 1.17)	0.33	0.45
Tenericutes;RF3;ML615J-28	0.61	0.02 (-0.06, 0.11)	1.02 (0.93, 1.10)	0.60	0.67

[1] FDR adjusted p value.

Supplementary Table S2 continued

Taxa	Mean	Log2FC (95% CI)	FC (95% CI)	p	q[1]
Phylum;Class;Order;Family					
Actinobacteria;Actinobacteria;Actinomycetales;Actinomycetaceae	966.83	0.10 (-0.03, 0.23)	1.07 (0.94, 1.20)	0.13	0.30
Actinobacteria;Actinobacteria;Actinomycetales;Corynebacteriaceae	214.95	-0.08 (-0.20, 0.04)	0.95 (0.82, 1.07)	0.19	0.33
Actinobacteria;Actinobacteria;Actinomycetales;Micrococcaceae	1474.45	-0.12 (-0.24, 0.00)	0.92 (0.80, 1.04)	0.05	0.18
Actinobacteria;Actinobacteria;Bifidobacteriales;Bifidobacteriaceae	54.59	0.06 (-0.08, 0.20)	1.04 (0.90, 1.19)	0.38	0.55
Actinobacteria;Coriobacteriia;Coriobacteriales;Coriobacteriaceae	180.45	0.02 (-0.11, 0.14)	1.01 (0.89, 1.14)	0.79	0.79
Bacteroidetes;Bacteroidia;Bacteroidales;[Paraprevotellaceae]	769.04	0.20 (0.08, 0.31)	1.15 (1.03, 1.26)	0.00	0.03
Bacteroidetes;Bacteroidia;Bacteroidales;Porphyromonadaceae	873.11	-0.03 (-0.14, 0.08)	0.98 (0.87, 1.09)	0.58	0.65
Bacteroidetes;Bacteroidia;Bacteroidales;Prevotellaceae	6264.20	0.07 (-0.03, 0.17)	1.05 (0.95, 1.15)	0.18	0.33
Bacteroidetes;Flavobacteriia;Flavobacteriales;[Weeksellaceae]	115.73	-0.08 (-0.20, 0.04)	0.94 (0.82, 1.06)	0.17	0.32
Bacteroidetes;Flavobacteriia;Flavobacteriales;Flavobacteriaceae	147.53	-0.15 (-0.26, -0.04)	0.90 (0.79, 1.01)	0.01	0.07
Chloroflexi;Anaerolineae;Anaerolineales;Anaerolinaceae	0.45	0.03 (-0.10, 0.15)	1.02 (0.90, 1.14)	0.68	0.73
Firmicutes;Bacilli;Bacillales;Bacillaceae	4.36	-0.06 (-0.20, 0.08)	0.96 (0.82, 1.10)	0.42	0.55
Firmicutes;Bacilli;Bacillales;Staphylococcaceae	1.37	-0.11 (-0.24, 0.02)	0.93 (0.79, 1.06)	0.10	0.27
Firmicutes;Bacilli;Gemellales;Gemellaceae	1149.33	-0.14 (-0.24, -0.04)	0.91 (0.81, 1.01)	0.01	0.07
Firmicutes;Bacilli;Lactobacillales;Aerococcaceae	82.61	-0.05 (-0.19, 0.09)	0.97 (0.82, 1.11)	0.49	0.60
Firmicutes;Bacilli;Lactobacillales;Carnobacteriaceae	33.25	-0.04 (-0.18, 0.09)	0.97 (0.84, 1.11)	0.55	0.65
Firmicutes;Bacilli;Lactobacillales;Enterococcaceae	1.17	-0.06 (-0.20, 0.08)	0.96 (0.82, 1.10)	0.40	0.55
Firmicutes;Bacilli;Lactobacillales;Lactobacillaceae	70.86	0.17 (0.05, 0.30)	1.13 (1.00, 1.26)	0.01	NA
Firmicutes;Bacilli;Lactobacillales;Streptococcaceae	14602.46	-0.13 (-0.22, -0.04)	0.91 (0.82, 1.00)	0.00	0.06
Firmicutes;Clostridia;Clostridiales;[Mogibacteriaceae]	118.31	0.14 (0.03, 0.25)	1.10 (0.99, 1.21)	0.01	0.07
Firmicutes;Clostridia;Clostridiales;[Tissierellaceae]	108.59	0.15 (0.03, 0.27)	1.11 (0.99, 1.23)	0.02	0.09
Firmicutes;Clostridia;Clostridiales;Eubacteriaceae	1.39	-0.05 (-0.18, 0.07)	0.97 (0.84, 1.09)	0.43	0.55
Firmicutes;Clostridia;Clostridiales;Lachnospiraceae	430.07	0.04 (-0.05, 0.13)	1.03 (0.94, 1.12)	0.37	0.55
Firmicutes;Clostridia;Clostridiales;Peptococcaceae	13.50	0.03 (-0.11, 0.17)	1.02 (0.88, 1.16)	0.69	0.73
Firmicutes;Clostridia;Clostridiales;Peptostreptococcaceae	134.94	0.10 (-0.02, 0.22)	1.07 (0.95, 1.20)	0.11	0.27
Firmicutes;Clostridia;Clostridiales;Veillonellaceae	3455.51	0.07 (-0.03, 0.17)	1.05 (0.95, 1.15)	0.17	0.32
Firmicutes;Erysipelotrichi;Erysipelotrichales;Erysipelotrichaceae	69.92	0.10 (-0.01, 0.22)	1.07 (0.96, 1.19)	0.08	0.25
Fusobacteria;Fusobacteriia;Fusobacteriales;Fusobacteriaceae	946.26	-0.01 (-0.10, 0.07)	0.99 (0.91, 1.08)	0.75	0.77
Fusobacteria;Fusobacteriia;Fusobacteriales;Leptotrichiaceae	698.28	0.03 (-0.07, 0.12)	1.02 (0.92, 1.11)	0.58	0.65
Proteobacteria;Betaproteobacteria;Burkholderiales;Burkholderiaceae	185.81	-0.08 (-0.22, 0.06)	0.95 (0.81, 1.09)	0.28	0.44
Proteobacteria;Betaproteobacteria;Burkholderiales;Comamonadaceae	4.62	0.07 (-0.06, 0.21)	1.05 (0.92, 1.19)	0.29	NA
Proteobacteria;Betaproteobacteria;Neisseriales;Neisseriaceae	2158.50	-0.09 (-0.22, 0.03)	0.94 (0.81, 1.06)	0.15	0.32
Proteobacteria;Deltaproteobacteria;Desulfobacterales;Desulfobulbaceae	0.91	0.14 (0.02, 0.27)	1.10 (0.98, 1.23)	0.02	0.12
Proteobacteria;Epsilonproteobacteria;Campylobacterales;Campylobacteraceae	160.04	-0.03 (-0.12, 0.05)	0.98 (0.89, 1.06)	0.43	0.55
Proteobacteria;Epsilonproteobacteria;Campylobacterales;Helicobacteraceae	0.61	0.07 (-0.02, 0.16)	1.05 (0.96, 1.14)	0.14	0.32
Proteobacteria;Gammaproteobacteria;Cardiobacteriales;Cardiobacteriaceae	20.94	-0.18 (-0.31, -0.04)	0.89 (0.75, 1.02)	0.01	0.07
Proteobacteria;Gammaproteobacteria;Pasteurellales;Pasteurellaceae	3783.97	-0.11 (-0.21, -0.01)	0.93 (0.82, 1.03)	0.04	0.15
Proteobacteria;Gammaproteobacteria;Pseudomonadales;Moraxellaceae	8.14	-0.02 (-0.13, 0.09)	0.98 (0.87, 1.09)	0.67	NA
Spirochaetes;Spirochaetes;Spirochaetales;Spirochaetaceae	201.93	0.12 (-0.02, 0.25)	1.08 (0.95, 1.22)	0.09	0.26

[1] FDR adjusted p value.

Supplementary Table S2 continued

Taxa	Mean	Log2FC (95% CI)	FC (95% CI)	p	q[1]
Phylum;Class;Order;Family;Genus					
Actinobacteria;Actinobacteria;Actinomycetales;Actinomycetaceae;Actinomyces	933.03	0.13 (-0.01, 0.28)	1.10 (0.95, 1.24)	0.08	0.17
Actinobacteria;Actinobacteria;Actinomycetales;Actinomycetaceae;Mobiluncus	1.86	0.12 (-0.06, 0.30)	1.09 (0.91, 1.27)	0.18	NA
Actinobacteria;Actinobacteria;Actinomycetales;Corynebacteriaceae;Corynebacterium	210.58	-0.12 (-0.25, 0.02)	0.92 (0.79, 1.06)	0.09	0.19
Actinobacteria;Actinobacteria;Actinomycetales;Micrococcaceae;Rothia	1497.42	-0.13 (-0.27, 0.01)	0.91 (0.77, 1.05)	0.06	0.15
Actinobacteria;Actinobacteria;Bifidobacteriales;Bifidobacteriaceae;Bifidobacterium	11.14	0.46 (0.27, 0.64)	1.37 (1.19, 1.56)	0.00	0.00
Actinobacteria;Actinobacteria;Bifidobacteriales;Bifidobacteriaceae;Scardovia	34.87	-0.10 (-0.27, 0.08)	0.94 (0.76, 1.11)	0.28	0.44
Actinobacteria;Coriobacteriia;Coriobacteriales;Coriobacteriaceae;Atopobium	157.99	0.03 (-0.10, 0.17)	1.02 (0.89, 1.16)	0.64	0.76
Actinobacteria;Coriobacteriia;Coriobacteriales;Coriobacteriaceae;Slackia	0.48	0.02 (-0.15, 0.19)	1.01 (0.84, 1.18)	0.85	0.87
Bacteroidetes;Bacteroidia;Bacteroidales;[Paraprevotellaceae];[Prevotella]	737.22	0.21 (0.09, 0.34)	1.16 (1.04, 1.28)	0.00	0.02
Bacteroidetes;Bacteroidia;Bacteroidales;Porphyromonadaceae;Paludibacter	24.48	-0.22 (-0.38, -0.06)	0.86 (0.70, 1.02)	0.01	0.06
Bacteroidetes;Bacteroidia;Bacteroidales;Porphyromonadaceae;Porphyromonas	815.76	-0.01 (-0.13, 0.12)	1.00 (0.87, 1.12)	0.93	0.93
Bacteroidetes;Bacteroidia;Bacteroidales;Porphyromonadaceae;Tannerella	36.08	-0.06 (-0.19, 0.06)	0.96 (0.83, 1.08)	0.30	0.44
Bacteroidetes;Bacteroidia;Bacteroidales;Prevotellaceae;Prevotella	5961.24	0.08 (-0.02, 0.19)	1.06 (0.96, 1.16)	0.12	0.22
Bacteroidetes;Flavobacteriia;Flavobacteriales;Flavobacteriaceae;Capnocytophaga	147.68	-0.16 (-0.29, -0.04)	0.89 (0.77, 1.02)	0.01	0.07
Chloroflexi;Anaerolineae;Anaerolineales;Anaerolinaceae;SHD-231	0.42	0.04 (-0.14, 0.21)	1.03 (0.85, 1.20)	0.69	0.80
Firmicutes;Bacilli;Bacillales;Bacillaceae;Bacillus	4.40	-0.07 (-0.25, 0.10)	0.95 (0.78, 1.13)	0.42	0.57
Firmicutes;Bacilli;Bacillales;Staphylococcaceae;Staphylococcus	1.29	-0.15 (-0.34, 0.03)	0.90 (0.72, 1.08)	0.11	0.21
Firmicutes;Bacilli;Gemellales;Gemellaceae;Gemella	20.59	-0.07 (-0.20, 0.07)	0.95 (0.82, 1.09)	0.33	0.47
Firmicutes;Bacilli;Lactobacillales;Carnobacteriaceae;Granulicatella	35.17	-0.07 (-0.23, 0.09)	0.95 (0.79, 1.11)	0.38	0.52
Firmicutes;Bacilli;Lactobacillales;Enterococcaceae;Vagococcus	1.21	-0.06 (-0.23, 0.10)	0.96 (0.79, 1.12)	0.45	0.59
Firmicutes;Bacilli;Lactobacillales;Lactobacillaceae;Lactobacillus	62.45	0.25 (0.07, 0.43)	1.19 (1.01, 1.37)	0.01	NA
Firmicutes;Bacilli;Lactobacillales;Streptococcaceae;Streptococcus	14718.21	-0.15 (-0.25, -0.05)	0.90 (0.80, 1.00)	0.00	0.05
Firmicutes;Clostridia;Clostridiales;[Mogibacteriaceae];Anaerovorax	12.12	0.14 (-0.04, 0.33)	1.10 (0.92, 1.29)	0.13	0.24
Firmicutes;Clostridia;Clostridiales;[Mogibacteriaceae];Mogibacterium	37.92	0.17 (0.05, 0.29)	1.12 (1.01, 1.24)	0.01	0.05
Firmicutes;Clostridia;Clostridiales;[Tissierellaceae];Parvimonas	101.51	0.18 (0.04, 0.31)	1.13 (0.99, 1.27)	0.01	0.07
Firmicutes;Clostridia;Clostridiales;Eubacteriaceae;Pseudoramibacter_Eubacterium	1.32	-0.05 (-0.23, 0.13)	0.96 (0.78, 1.14)	0.55	0.67
Firmicutes;Clostridia;Clostridiales;Lachnospiraceae;Butyrivibrio	5.71	0.09 (-0.09, 0.26)	1.06 (0.89, 1.24)	0.32	0.47
Firmicutes;Clostridia;Clostridiales;Lachnospiraceae;Catonella	39.46	0.12 (-0.01, 0.25)	1.09 (0.96, 1.21)	0.07	0.16
Firmicutes;Clostridia;Clostridiales;Lachnospiraceae;Moryella	52.66	0.02 (-0.10, 0.15)	1.02 (0.89, 1.14)	0.71	0.80
Firmicutes;Clostridia;Clostridiales;Lachnospiraceae;Oribacterium	170.52	0.11 (-0.01, 0.23)	1.08 (0.96, 1.20)	0.06	0.15
Firmicutes;Clostridia;Clostridiales;Peptococcaceae;Peptococcus	12.87	0.05 (-0.11, 0.21)	1.03 (0.87, 1.20)	0.55	0.67
Firmicutes;Clostridia;Clostridiales;Peptostreptococcaceae;Filifactor	27.57	0.06 (-0.11, 0.24)	1.05 (0.87, 1.22)	0.48	0.61
Firmicutes;Clostridia;Clostridiales;Peptostreptococcaceae;Peptostreptococcus	87.22	0.18 (0.03, 0.33)	1.13 (0.98, 1.28)	0.02	0.08
Firmicutes;Clostridia;Clostridiales;Veillonellaceae;Dialister	53.65	0.12 (0.00, 0.24)	1.09 (0.97, 1.21)	0.04	0.13
Firmicutes;Clostridia;Clostridiales;Veillonellaceae;Megasphaera	179.09	0.15 (0.00, 0.29)	1.11 (0.96, 1.25)	0.05	0.14
Firmicutes;Clostridia;Clostridiales;Veillonellaceae;Schwartzia	7.78	0.19 (0.04, 0.35)	1.14 (0.99, 1.30)	0.02	0.08
Firmicutes;Clostridia;Clostridiales;Veillonellaceae;Selenomonas	136.12	0.20 (0.07, 0.33)	1.15 (1.01, 1.28)	0.00	0.05
Firmicutes;Clostridia;Clostridiales;Veillonellaceae;Veillonella	2903.85	0.07 (-0.03, 0.18)	1.05 (0.95, 1.16)	0.17	0.28
Firmicutes;Erysipelotrichi;Erysipelotrichales;Erysipelotrichaceae;Bulleidia	65.36	0.13 (0.01, 0.26)	1.10 (0.97, 1.22)	0.04	0.11
Firmicutes;Erysipelotrichi;Erysipelotrichales;Erysipelotrichaceae;Sharpea	1.52	0.11 (-0.07, 0.29)	1.08 (0.90, 1.26)	0.25	NA
Fusobacteria;Fusobacteriia;Fusobacteriales;Fusobacteriaceae;Fusobacterium	913.06	-0.01 (-0.10, 0.07)	0.99 (0.90, 1.08)	0.74	0.82
Fusobacteria;Fusobacteriia;Fusobacteriales;Leptotrichiaceae;Leptotrichia	619.90	0.01 (-0.09, 0.11)	1.01 (0.91, 1.11)	0.85	0.87
Proteobacteria;Betaproteobacteria;Burkholderiales;Burkholderiaceae;Lautropia	200.38	-0.10 (-0.27, 0.07)	0.93 (0.76, 1.11)	0.26	NA
Proteobacteria;Betaproteobacteria;Neisseriales;Neisseriaceae;Eikenella	25.81	-0.19 (-0.34, -0.04)	0.88 (0.73, 1.03)	0.01	NA
Proteobacteria;Betaproteobacteria;Neisseriales;Neisseriaceae;Kingella	27.15	-0.11 (-0.26, 0.04)	0.93 (0.77, 1.08)	0.14	0.25
Proteobacteria;Betaproteobacteria;Neisseriales;Neisseriaceae;Neisseria	2152.22	-0.09 (-0.24, 0.06)	0.94 (0.79, 1.09)	0.24	0.37
Proteobacteria;Deltaproteobacteria;Desulfobacterales;Desulfobulbaceae;Desulfobulbus	0.86	0.21 (0.03, 0.39)	1.16 (0.98, 1.34)	0.02	0.08
Proteobacteria;Epsilonproteobacteria;Campylobacterales;Campylobacteraceae;Campylobacter	152.07	-0.01 (-0.09, 0.07)	0.99 (0.91, 1.07)	0.85	0.87
Proteobacteria;Gammaproteobacteria;Cardiobacteriales;Cardiobacteriaceae;Cardiobacterium	20.86	-0.21 (-0.36, -0.06)	0.87 (0.71, 1.02)	0.01	0.06
Proteobacteria;Gammaproteobacteria;Pasteurellales;Pasteurellaceae;Actinobacillus	24.67	-0.17 (-0.33, 0.00)	0.89 (0.72, 1.06)	0.05	0.14
Proteobacteria;Gammaproteobacteria;Pasteurellales;Pasteurellaceae;Aggregatibacter	332.36	-0.17 (-0.31, -0.03)	0.89 (0.75, 1.03)	0.02	0.08
Proteobacteria;Gammaproteobacteria;Pasteurellales;Pasteurellaceae;Haemophilus	3479.38	-0.13 (-0.25, -0.01)	0.92 (0.80, 1.03)	0.04	0.11
Proteobacteria;Gammaproteobacteria;Pseudomonadales;Moraxellaceae;Enhydrobacter	2.04	-0.02 (-0.19, 0.14)	0.98 (0.82, 1.15)	0.77	0.84
Proteobacteria;Gammaproteobacteria;Pseudomonadales;Moraxellaceae;Moraxella	6.22	-0.02 (-0.15, 0.10)	0.98 (0.86, 1.11)	0.70	NA
Spirochaetes;Spirochaetes;Spirochaetales;Spirochaetaceae;Treponema	189.02	0.16 (0.01, 0.32)	1.12 (0.97, 1.28)	0.04	0.11
Synergistetes;Synergistia;Synergistales;Dethiosulfovibrionaceae;TG5	22.87	0.13 (-0.04, 0.30)	1.09 (0.93, 1.26)	0.13	0.24
Tenericutes;Mollicutes;Mycoplasmatales;Mycoplasmataceae;Mycoplasma	17.25	0.19 (0.02, 0.37)	1.14 (0.97, 1.32)	0.03	0.11

[1] FDR adjusted p value.

Supplementary Table S3. Relative abundances of selected taxa of the oral microbiome as exposure to incense increases according to smoking habits.

Taxa[1]	All participants (n= 303)				Nonsmokers (n= 190)				Smokers (n= 83)			
	Mean	log2FC (95% CI)	p	q[2]	Mean	log2FC (95% CI)	p	q[2]	Mean	log2FC (95% CI)	p	q[2]
Class												
Firmicutes;Bacilli	18098.32	-0.12 (-0.21, -0.03)	0.01	0.07	17947.01	-0.09 (-0.18, 0.01)	0.09	0.50	16805.77	-0.10 (-0.25, 0.04)	0.17	0.45
Proteobacteria;Deltaproteobacteria	0.90	0.12 (0.02, 0.22)	0.02	0.09	0.82	0.03 (-0.03, 0.10)	0.36	0.60	1.33	0.15 (0.03, 0.28)	0.02	0.34
Tenericutes;Mollicutes	26.83	0.17 (0.05, 0.29)	0.01	0.07	26.07	0.11 (0.01, 0.22)	0.03	NA	33.37	0.06 (-0.11, 0.22)	0.51	0.81
Order												
Bacteroidetes;Bacteroidia;Bacteroidales	8050.98	0.12 (0.04, 0.21)	0.01	0.09	7899.31	0.16 (0.06, 0.26)	1.84E-03	0.04	8746.75	-0.03 (-0.18, 0.12)	0.71	0.80
Firmicutes;Clostridia;Clostridiales	4329.04	0.11 (0.03, 0.19)	0.01	0.09	4109.17	0.12 (0.02, 0.22)	0.02	0.18	4773.20	0.05 (-0.09, 0.18)	0.51	0.79
Firmicutes;Erysipelotrichi;Erysipelotrichales	72.86	0.13 (0.02, 0.25)	0.02	9.70E-02	72.51	0.12 (-0.01, 0.24)	0.07	0.28	76.17	-0.03 (-0.20, 0.14)	0.73	0.80
Proteobacteria;Deltaproteobacteria;Desulfobacterales	0.93	0.14 (0.03, 0.26)	0.01	0.09	0.85	0.05 (-0.04, 0.14)	0.29	0.44	1.33	0.14 (0.01, 0.26)	0.03	0.32
Tenericutes;Mollicutes;Mycoplasmatales	18.81	0.16 (0.03, 0.29)	0.02	0.09	16.57	0.10 (-0.02, 0.23)	0.11	NA	27.72	0.06 (-0.10, 0.22)	0.47	0.79
Family												
Bacteroidetes;Bacteroidia;Bacteroidales;[Paraprevotellaceae]	769.04	0.20 (0.08, 0.31)	7.26E-04	0.03	712.28	0.23 (0.10, 0.37)	8.66E-04	0.03	918.18	0.08 (-0.13, 0.29)	0.44	0.85
Bacteroidetes;Flavobacteriia;Flavobacteriales;Flavobacteriaceae	147.53	-0.15 (-0.26, -0.04)	0.01	0.07	160.07	-0.15 (-0.29, -0.01)	0.03	0.17	132.49	-0.09 (-0.29, 0.11)	0.40	0.85
Firmicutes;Bacilli;Gemellales;Gemellaceae	1149.33	-0.14 (-0.24, -0.04)	0.01	0.07	1217.20	-0.18 (-0.30, -0.06)	4.02E-03	0.06	957.78	0.06 (-0.13, 0.24)	0.54	0.85
Firmicutes;Bacilli;Lactobacillales;Streptococcaceae	14602.46	-0.13 (-0.22, -0.04)	3.21E-03	0.06	14239.38	-0.11 (-0.22, 0.00)	0.05	0.24	14054.69	-0.19 (-0.36, -0.02)	0.03	0.28
Firmicutes;Clostridia;Clostridiales;[Mogibacteriaceae]	118.31	0.14 (0.03, 0.25)	0.01	0.07	109.48	0.15 (0.02, 0.29)	0.02	0.16	140.52	-0.09 (-0.28, 0.10)	0.35	0.85
Firmicutes;Clostridia;Clostridiales;[Tissierellaceae]	108.59	0.15 (0.03, 0.27)	0.02	0.09	103.97	0.16 (0.01, 0.31)	0.03	0.17	122.63	-0.07 (-0.28, 0.14)	0.52	0.85
Proteobacteria;Gammaproteobacteria;Cardiobacteriales;Cardiobacteriaceae	20.94	-0.18 (-0.31, -0.04)	0.01	0.07	22.89	-0.22 (-0.38, -0.07)	0.01	NA	18.16	0.03 (-0.19, 0.25)	0.79	0.96
Genus												
Actinobacteria;Actinobacteria;Bifidobacteriales;Bifidobacteriaceae;Bifidobacterium	11.14	0.46 (0.27, 0.64)	1.15E-06	5.88E-05	5.68	0.30 (0.12, 0.48)	8.70E-04	0.02	22.18	0.26 (0.05, 0.47)	0.01	0.24
Bacteroidetes;Bacteroidia;Bacteroidales;[Paraprevotellaceae];[Prevotella]	737.22	0.21 (0.09, 0.34)	7.38E-04	0.02	689.17	0.24 (0.10, 0.39)	8.59E-04	0.02	882.80	0.14 (-0.07, 0.35)	0.18	0.69
Bacteroidetes;Bacteroidia;Bacteroidales;Porphyromonadaceae;Paludibacter	24.48	-0.22 (-0.38, -0.06)	0.01	0.06	23.71	-0.29 (-0.47, -0.12)	1.07E-03	0.02	31.35	-0.04 (-0.27, 0.19)	0.74	0.97
Bacteroidetes;Flavobacteriia;Flavobacteriales;Flavobacteriaceae;Capnocytophaga	147.68	-0.16 (-0.29, -0.04)	0.01	0.07	161.37	-0.15 (-0.29, 0.00)	0.06	0.21	132.26	-0.09 (-0.30, 0.11)	0.37	0.97
Firmicutes;Bacilli;Lactobacillales;Streptococcaceae;Streptococcus	14718.21	-0.15 (-0.25, -0.05)	4.10E-03	0.05	14464.93	-0.12 (-0.24, 0.01)	0.06	0.21	13947.87	-0.16 (-0.34, 0.01)	0.07	0.43
Firmicutes;Clostridia;Clostridiales;[Mogibacteriaceae];Mogibacterium	37.92	0.17 (0.05, 0.29)	0.01	0.05	35.55	0.16 (0.02, 0.30)	0.02	0.14	40.90	-0.04 (-0.24, 0.16)	0.70	0.97
Firmicutes;Clostridia;Clostridiales;[Tissierellaceae];Parvimonas	101.51	0.18 (0.04, 0.31)	0.01	0.07	98.13	0.16 (0.00, 0.32)	0.05	0.21	115.59	-0.04 (-0.25, 0.17)	0.69	0.97
Firmicutes;Clostridia;Clostridiales;Peptostreptococcaceae;Peptostreptococcus	87.22	0.18 (0.03, 0.33)	0.02	0.08	90.86	0.17 (0.00, 0.33)	0.04	0.21	82.66	0.01 (-0.22, 0.24)	0.95	0.97
Firmicutes;Clostridia;Clostridiales;Veillonellaceae;Schwartzia	7.78	0.19 (0.04, 0.35)	0.02	0.08	7.16	0.17 (-0.01, 0.35)	0.06	0.21	8.52	0.17 (-0.06, 0.39)	0.14	0.64
Firmicutes;Clostridia;Clostridiales;Veillonellaceae;Selenomonas	136.12	0.20 (0.07, 0.33)	3.30E-03	0.05	124.26	0.16 (0.00, 0.31)	0.05	0.21	161.91	0.33 (0.13, 0.53)	1.25E-03	0.06
Proteobacteria;Deltaproteobacteria;Desulfobacterales;Desulfobulbaceae;Desulfobulbus	0.86	0.21 (0.03, 0.39)	0.02	0.08	0.74	0.06 (-0.10, 0.22)	0.46	0.62	1.28	0.22 (0.03, 0.41)	0.03	0.32
Proteobacteria;Gammaproteobacteria;Cardiobacteriales;Cardiobacteriaceae;Cardiobacterium	20.86	-0.21 (-0.36, -0.06)	0.01	0.06	23.19	-0.25 (-0.42, -0.08)	3.89E-03	NA	17.68	0.04 (-0.18, 0.26)	0.73	NA
Proteobacteria;Gammaproteobacteria;Pasteurellales;Pasteurellaceae;Aggregatibacter	332.36	-0.17 (-0.31, -0.03)	0.02	0.08	348.84	-0.10 (-0.26, 0.06)	0.23	0.46	308.35	-0.19 (-0.41, 0.03)	0.10	NA

[1] Only those taxa that had a significantly differential abundance with q<0.1 and a Cook's distance <10 for the trend analysis are shown.

[2] FDR adjusted p value implemented independently at each level (i.e. phylum, class …).

Supplementary Table S4. Differences in mean abundances between groups represented as percentage change in the pairwise comparisons.

Genus[1]	Never - Occasional	Occasional - Frequent	Frequent - Daily
Actinobacteria;Actinobacteria;Bifidobacteriales;Bifidobacteriaceae;Bifidobacterium	-4.21	93.66	154.28
Bacteroidetes;Bacteroidia;Bacteroidales;[Paraprevotellaceae];[Prevotella]	49.41	18.11	11.43
Bacteroidetes;Bacteroidia;Bacteroidales;Porphyromonadaceae;Paludibacter	-58.48	-0.57	-2.49
Bacteroidetes;Flavobacteriia;Flavobacteriales;Flavobacteriaceae;Capnocytophaga	-23.21	-5.18	-14.28
Firmicutes;Bacilli;Lactobacillales;Streptococcaceae;Streptococcus	-21.16	-2.65	-11.69
Firmicutes;Clostridia;Clostridiales;[Mogibacteriaceae];Mogibacterium	66.59	10.66	5.61
Firmicutes;Clostridia;Clostridiales;[Tissierellaceae];Parvimonas	77.06	15.12	0.66
Firmicutes;Clostridia;Clostridiales;Peptostreptococcaceae;Peptostreptococcus	82.47	47.99	-24.76
Firmicutes;Clostridia;Clostridiales;Veillonellaceae;Schwartzia	-7.31	17.23	30.63
Firmicutes;Clostridia;Clostridiales;Veillonellaceae;Selenomonas	-21.68	33.56	24.06
Proteobacteria;Deltaproteobacteria;Desulfobacterales;Desulfobulbaceae;Desulfobulbus	12.77	20.75	112.50
Proteobacteria;Gammaproteobacteria;Cardiobacteriales;Cardiobacteriaceae;Cardiobacterium	-41.68	-0.19	-10.51
Proteobacteria;Gammaproteobacteria;Pasteurellales;Pasteurellaceae;Aggregatibacter	-5.14	-21.64	-6.13

* Differences in mean abundances are represented as percentage change relative to the incense group in the comparison of interest. Negative values indicate depletion while positive values indicate enrichment.

[1] Only those genera that had a significantly differential abundance with q<0.1 and a Cook's distance <10 for the trend analysis are shown.
